# Elucidation of the Molecular Mechanisms Underlying Sorafenib-Induced Hepatotoxicity

**DOI:** 10.1155/2020/7453406

**Published:** 2020-05-14

**Authors:** Abdullah F. AlAsmari, Nemat Ali, Fawaz AlAsmari, Wael A. AlAnazi, Faleh Alqahtani, Metab Alharbi, Farraj M. Alotaibi, Abdullah A. Aldossari, Mohammed AlSwayyed, Mohammed M. Alanazi, Ali A. Alshamrani

**Affiliations:** ^1^Department of Pharmacology and Toxicology, College of Pharmacy, King Saud University, Riyadh 11451, Saudi Arabia; ^2^Department of Pathology, College of Medicine, King Saud University, Riyadh 11451, Saudi Arabia

## Abstract

Sorafenib is a small, orally-active multikinase inhibitor that is most frequently used for the management of renal cell carcinoma, hepatocellular carcinoma, and radioactive iodine-resistant thyroid carcinoma. However, recent reports have associated sorafenib with hepatotoxicity that can limit its clinical application, although the mechanism of hepatotoxicity is still to be elucidated. Thus, our study was designed to explore the molecular mechanisms underlying sorafenib-induced hepatotoxicity in an *in vivo* model. Twenty male adult Wistar rats were randomly placed into two groups; the first group received an oral dose of normal saline (vehicle), and the second received sorafenib (30 mg/kg) once daily for twenty-one consecutive days. After twenty-one days, liver tissues and blood samples were used for gene expression, protein expression, and biochemical analysis. Sorafenib treatment resulted in markedly increased levels of alanine aminotransferase and alkaline phosphatase, which indicate the presence of liver damage. Additionally, sorafenib administration induced the inflammatory and oxidative stress marker NF-*κ*B-p65, while antioxidant enzymes were attenuated. Moreover, sorafenib caused upregulation of both gene and protein for the apoptotic markers cleaved Caspase-3, Bax, and Bid, and downregulation of the antiapoptotic protein Bcl-2. In conclusion, our findings suggest that sorafenib administration is associated with hepatotoxicity, which might be due to the activation of oxidative stress and apoptotic pathways.

## 1. Introduction

Tyrosine kinases (TKs) are key enzymes that play essential roles in a variety of biological activities, especially proliferation, differentiation, and survival of cells. Upon activation by extracellular signaling molecules, TKs activate several signaling pathways that are actively involved in diverse aspects of cellular physiology. Recently, it has been reported that mutations in TKs or overexpression of defective TKs are linked to cancer initiation, progression, and metastasis [1–3]. Because TKs are involved in the process of cancer development, a new class of drugs known as tyrosine kinase inhibitors (TKIs) was developed to block the activation of TKs and the signaling pathways downstream [1]. Currently, TKIs are used to treat a number of types of cancers [2–4].

Drugs that inhibit the activities of multiple TKs are known as multikinase inhibitors (MKIs). One of the most commonly used MKIs is sorafenib (SORA), a small molecule that is orally active and has anticancer and antiangiogenic activities [5, 6]. SORA was designed as a strong candidate for the inactivation of the unregulated Raf/MEK/ERK signaling cascade, which has been reported as a key factor in several cancers and other diseases [7]. SORA has been reported to target B-RAF, C-RAF, platelet-derived growth factor receptor- (PDGFR-) *β*, vascular endothelial growth factor receptors (VEGFR-1, -2, -3), c-kit, RET, and Fms-like tyrosine kinase-3 (Flt-3). Furthermore, SORA has the ability to hinder cancer growth, progression, metastasis, and angiogenesis, as well as to downregulate mechanisms that prevent cancers from being subject to apoptosis [8, 9]. Accordingly, SORA was approved for the use in renal cell carcinoma (RCC) and in the management of hepatocellular carcinoma (HCC) [10–12].

Although SORA is indicated for the treatment of different types of cancers and has been documented to improve survival in cancer patients, a spectrum of side effects has been reported, including loss of appetite, high blood pressure, diarrhea, hand-foot syndrome, acne, and other health-related problems [13, 14]. Recently, Zhang and his colleagues investigated the possible hepatotoxic effects of 31 FDA-approved TKIs [15]. They reported that SORA was one of three TKIs that were hepatotoxic at their Cmax concentrations and concluded that mitochondrial toxicity may contribute to this hepatotoxicity [15]. An *in vitro* study has also suggested that SORA-induced apoptosis is realized through reactive oxygen species (ROS) generation, JNK/p38-MAPK activation, and Bax translocation [16]. In addition, it has been shown that SORA treatment induced the activity of NF-*κ*B-p65, which is reported to be induced in response to oxidative stress and inflammation leading to the development of HCC [17–19].

It is possible that SORA-induced hepatotoxicity is mediated through alterations in oxidative stress and apoptosis. However, no report to date has confirmed this; the precise mechanisms underlying SORA-induced hepatotoxicity remain unclear. In one study, Amemiya and his colleague studied the toxic effects of SORA and sunitinib using the mouse model. In their study, they found that 14 days of treatment of sunitinib (26.7 mg/kg), but not SORA (20.6 mg/kg), resulted in hepatotoxicity. Therefore, the current study aimed at examining the association of chronic treatment of SORA with liver toxicity in an *in vivo* model. Our results confirmed that chronic treatment with SORA induced liver toxicity, which manifested in terms of elevated liver enzymes, elevated oxidative stress markers, and dysregulated antioxidant mechanisms.

## 2. Methodology

### 2.1. Animals

Animals used in our study were taken from the animal facility at the College of Pharmacy, King Saud University and maintained in conditions regulated for temperature and humidity (23°C and 12 h. light/dark cycles) with free access to drinking water and standard diet. Animals were housed in clean cages and left to acclimatize without disturbance for 10 days prior to the start of the experiments. The experimental protocols and procedures mentioned in our study were in compliance with the National Institutes of Health guidelines for the Care and Use of Laboratory Animals, and it is completely approved and accepted by the local institutional research ethics committee of King Saud University (KSU-SE-18-41).

### 2.2. Experimental Design and Treatment Protocol

Twenty male adult Wistar rats (weighing between 180 and 200 g) were used in our study and were randomly divided into two groups, with ten rats per group. Animals in group 1 (control) and group 2 (SORA), respectively, received equal doses of normal saline (0.9% NaCl P.O.) and sorafenib (30 mg/kg P.O.) once daily for 21 consecutive days [20]. Body weight was monitored daily during the study and the dose adjusted as needed.

At the end of the study, rats were fasted overnight and anesthetized by i.p. injection of ketamine/xylazine solution (ketamine 100 mg/kg and xylazine 10 mg/kg) [21], after which blood was collected directly from the hearts, and the plasma separated in order to measure liver enzymes and assess liver markers. In addition, liver tissues were harvested and washed immediately with cold phosphate-buffered saline (PBS) and directly kept in liquid nitrogen then stored at -80°C until the time of experiments. Thereafter, frozen liver tissues were used to conduct biochemical, protein expression, and gene expression analyses using commercially available kits according to their protocols.

### 2.3. Measurement of Plasma Markers

Plasma was obtained from whole blood samples by centrifugation for 5 minutes at 2000 g and 4°C. Then, the levels of cholesterol, triglycerides, high-density lipoprotein (HDL), low-density lipoprotein (LDL), very low-density lipoprotein (VLDL), alanine transaminase (ALT), aspartate aminotransferase (AST), alkaline phosphatase (ALP), bilirubin, albumin, and urea were measured using the automated Dimension® RXL MAX Integrated Chemistry System (Siemens, USA).

### 2.4. Western Blot Analysis

Total proteins were extracted from liver tissue by homogenizing the samples in cold lysis buffer (Thermo Scientific, USA) that was mixed with protease and phosphatase inhibitors (Thermo Scientific, USA). The resulting tissue homogenates were centrifuged, clear supernatants collected, and total proteins quantified using a Direct Detect® spectrometer (EMD Millipore, USA). After that, the protein lysates were mixed with 2x Laemmli buffer (Bio-Rad, USA) that was supplemented with *β*-mercaptoethanol (*β*ME). Then, protein lysates were heated at 95°C for five minutes. Thereafter, equal amounts of proteins were loaded in the wells of a 12% SDS-PAGE gel, resolved, and transferred to PVDF membranes using the Trans-Blot Turbo Transfer System (Bio-Rad, USA). Membranes were then blocked for one hour with 5% nonfat dry milk in tris-buffered saline (TBS) containing 0.1% Tween-20 at room temperature. After blocking, membranes were separately incubated at 4°C on a rocker with primary antibodies specific to the protein of interest; these were rabbit antisuperoxide dismutase-2 (SOD2) antibody (cat# A1340), anti-B-cell lymphoma-2 (Bcl-2) antibody (cat# A0208), anti-Bcl-2-associated X (Bax) antibody (cat# A12009), anticleaved Caspase-3 antibody (cat# A2156), and mouse anti-Glyceraldehyde 3-phosphate dehydrogenase (GAPDH) antibody (cat# AC001). All the primary antibodies were purchased from ABclonal Technology, USA, and used in dilution of 1 : 1000. Subsequently, the membranes were incubated with a suitable HRP-conjugated secondary antibody (Cell signaling technology, USA) for one hour. Finally, bands were developed using a chemiluminescence reagent (Merck Millipore, USA) and visualized using a ChemiDoc MP Imaging System (Bio-Rad, USA). The visualized blots were quantified and analyzed using ImageJ software [22].

### 2.5. Gene Expression Analysis by Real-Time Quantitative Polymerase Chain Reaction (RT-qPCR)

From liver tissues, total RNA was isolated using TRIzol ™ reagent (Thermo Scientific, USA) according to the manufacturer's guidance. The purity and concentrations of the isolated RNA were measured using a NanoDrop™ 8000 Spectrophotometer (Thermo Scientific, USA). Thereafter, cDNA was synthesized from the isolated RNA using a TaqMan™ Reverse Transcription kit (Thermo Scientific, USA). Changes in the expression of various genes were quantified by a Quant Studio 6 Flex real-time PCR System (Thermo Fisher Scientific, USA) using SYBR green master mix (Bimake, USA) with *β*-actin as the reference housekeeping gene. Relative expressions of mRNA were calculated by the *ΔΔ*Ct method [23]. Sequences of the forward and reverse primers (IDT, Belgium) that were used in the present study are as follow; Bax (5′-TAGCAAACTGGTGCTCAAGG-3′; 5′-TCTTGGATCCAGACAAGCAG-3′), Bid (5′-CCCACACTGGTGAGACAACT-3′; 5′-TGTCGTTCTCCATGTCCCTA-3′), NF-*κ*B-p65 (5′-CATGCGTTTCCGTTACAAGTGCGA-3′; 5′-TGGGTGCGTCTTAGTGGTATCTGT-3′), Bcl-2 (5′-CATGCGACCTCTGTTTGA-3′; 5′-GTTTCATGGTCCATCCTTG-3′), GPX-1 (5′-AGTTCGGACATCAGGAGAATGGCA-3′; 5′-TCACCATTCACCTCGCACTTCTCA-3′), HO-1 (5′-ACAGGGTGACAGAAGAGGCTAA-3′; 5′-CTGTGAGGGACTCTGGTCTTTG-3′), SOD (5′-TTCGTTTCCTGCGGCGGCTT-3′; 5′-TTCAGCACGCACACGGCCTT-3′), and *β*-actin (5′-CCAGATCATGTTTGAGACCTTCAA-3′; 5′-GTGGTACGACCAGAGGCATACA-3′).

### 2.6. Catalase Activity Measurement

Catalase enzyme activity was quantified using an EnzyChrom™ Catalase Assay Kit (cat# ECAT-100, BioAssay Systems, USA) as per the manufacturer's directions. Briefly, liver tissues were homogenized in cold PBS and then centrifuged at high speed for ten minutes. Afterwards, 10 *μ*l of samples was mixed with 90 *μ*l of 50 *μ*M H_2_O_2_ to initiate the catalase reaction and incubated at room temperature for 30 minutes. After that, 100 *μ*l of the detection reagent was added to the mixture and incubated for 10 minutes at room temperature. Then, the optical density (*Δ*OD) was measured at 550 nm.

### 2.7. Measurement of Glutathione (GSH)

Reduced glutathione (GSH) and oxidized glutathione (GSSG) were measured in liver tissue homogenates using an EnzyChrom™ Glutathione Assay Kit (BioAssay Systems, USA) according to the manufacturer's guidelines. In general, liver tissues were homogenized in cold buffer containing 1 mM EDTA and 50 mM phosphate (pH = 7) in the presence or absence of scavenger to measure GSSG and GSH, respectively. Then, tissue lysates were centrifuged at high speed for five minutes at 4°C and deproteinated using metaphosphoric acid. Afterwards, 200 *μ*l of samples was incubated with 100 *μ*l of detection reagent, which includes assay buffer, NADPH, DNTB, and glutathione reductase (GR). Finally, the optical density differences (*Δ*OD) were measured at 412 nm.

### 2.8. NAD^+^/NADH Measurement

NAD^+^/NADH was measured in liver tissue homogenates using an EnzyChrom™ NAD^+^/NADH Assay Kit (BioAssay Systems, USA) according to the manufacturer's procedure. Briefly, liver tissues were homogenized in NAD and NADH extraction buffers to determine NAD and NADH, respectively. Homogenates were heated at 60°C for five minutes, and then incubated with the opposite extraction buffer for neutralization. Next, the samples were centrifuged at 14,000 rpm for five minutes. After that, differences in optical density (*ΔΔ*OD) were measured at 565 nm.

### 2.9. Statistical Analysis

Results are presented as mean ± SD. All statistical analyses used a two-tailed student's *t*-test with the level of significance set at *P* < 0.05. Statistical analyses were achieved using GraphPad Prism 6.01 (CA, USA).

## 3. Results

### 3.1. Effect of Sorafenib on Liver Function Tests and Lipid Profile

**Figure 1:**
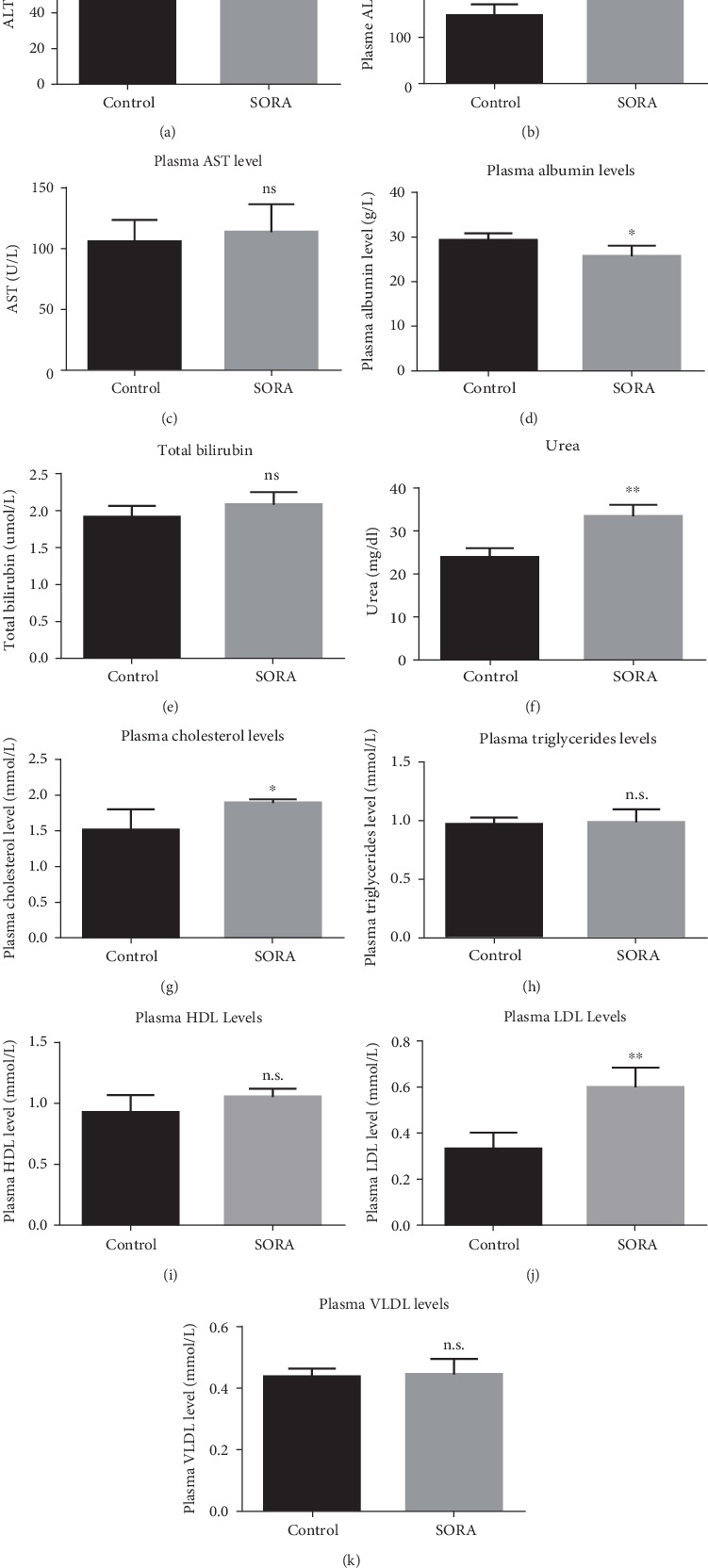
Plasma liver markers. Whole blood samples were used to separate plasma and measure the liver enzymes and liver markers. Data are presented as mean ± SD. Comparison between the control group and the sorafenib group is presented by ^∗^, where ^∗^*P* < 0.05, while ^∗∗^*P* < 0.01. n.s. means there were no significant changes (*P* > 0.05). SORA: sorafenib; ALT: alanine transaminase; ALP: alkaline phosphatase; AST: aspartate aminotransferase; HDL: high-density lipoprotein; LDL: low-density lipoprotein; VLDL: very low-density lipoprotein.

A number of plasma parameters are used clinically for the analysis of liver functions. To examine whether sorafenib (SORA) administration compromises liver function, we measured several liver function-associated enzymes (ALT, AST, and ALP), plasma proteins (albumin, bilirubin, and urea), and lipid profiles (cholesterol, triglycerides, HDL, LDL, and VLDL). We found that twenty-one days of oral SORA administration at a dose of 30 mg/kg significantly induced ALT (1.5 folds), ALP (1.8 folds), cholesterol (1.2 folds), LDL (1.8 folds), and urea (1.4 folds), whereas levels of AST, bilirubin, triglycerides, HDL, and VLDL were statistically comparable between groups (Figures 1(a)–1(k)). Furthermore, plasma albumin levels were noticeably lower in SORA-treated rats compared to control rats (1.1 folds) (Figure 1(d)). Taken together, these results suggest that SORA treatment is associated with liver damage.

### 3.2. Sorafenib Activates Oxidative Stress and Apoptotic Pathways

**Figure 2:**
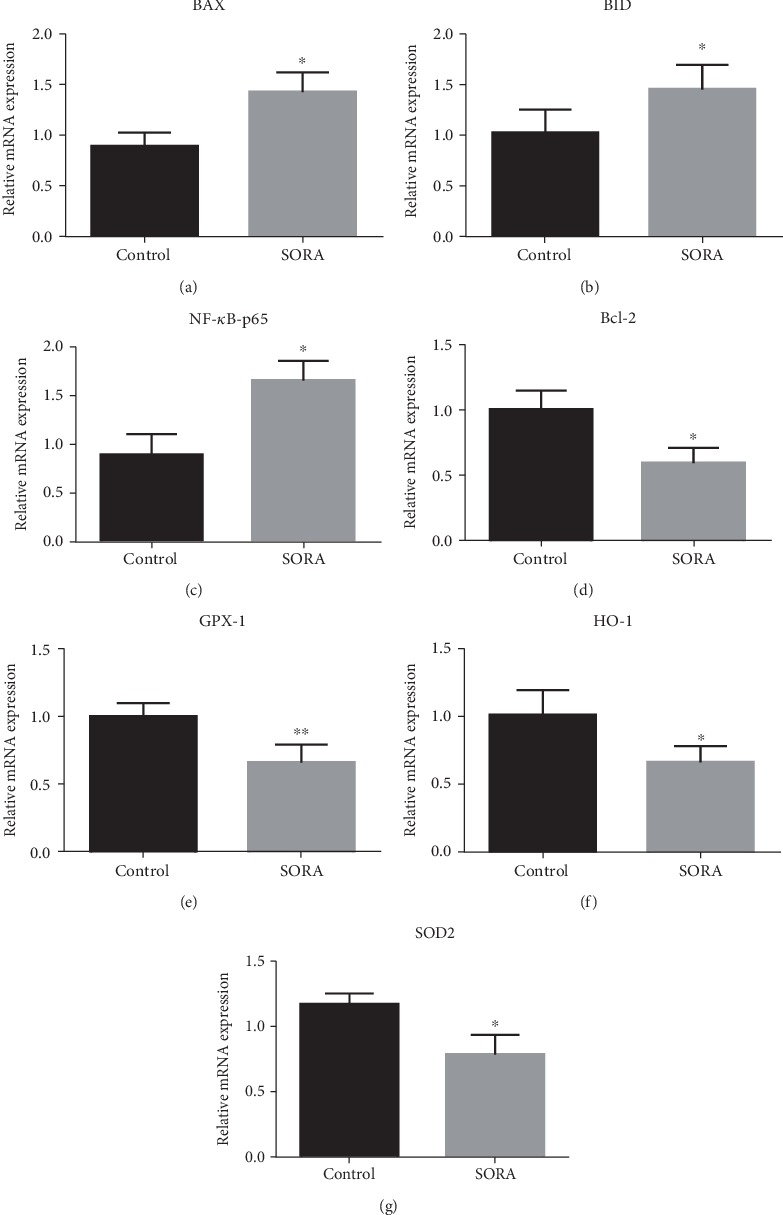
Gene expression analysis. RNA was isolated from the liver of different samples and was used to measure mRNA levels of different genes in each group (a–g) using Quantitative RT-QPCR. Data are presented as mean ± SD. Comparison between the control group and the sorafenib group is presented by ^∗^, where ^∗^*P* < 0.05, while ^∗∗^*P* < 0.01. SORA: sorafenib; BAX: Bcl-2 Associated X; BID: BH3 interacting-domain death agonist; NF-*κ*B-p65: Nuclear factor kappa B; Bcl-2: B-cell lymphoma-2; GPX-1: Glutathione peroxidase-1; HO-1: Heme Oxygenase-1; SOD2: superoxide dismutase-2.

To further investigate the mechanisms underlying SORA-induced hepatotoxicity, we measured the gene expression of multiple genes involved in apoptotic and oxidative stress pathways. Our mRNA analysis revealed that SORA administration considerably induced expression of proapoptotic genes (*BAX* and *BID*) (1.4 folds and 1.5 folds, respectively) and produced a noteworthy decrease in the levels of an antiapoptotic gene (*Bcl-2*) (2 folds) (Figures 2(a), 2(b), and 2(d), respectively). In addition, we detected a considerable increase in the expression of *NF-κB-p65* (1.6 folds) in the SORA-treated group (Figure 2(c)). Furthermore, we found that the antioxidant genes *GPX-1*, *HO-1*, and *SOD2* had considerably reduced expression in SORA-treated rats (1.5 folds, 1.5 folds, and 1.7 folds, respectively) (Figures 2(e)–2(g)). Together, these changes in the expression of proapoptotic and antiapoptotic genes suggest that SORA treatment is associated with liver toxicity.

### 3.3. Sorafenib Reduces the Levels of Antioxidant and Antiapoptotic Proteins

**Figure 3:**
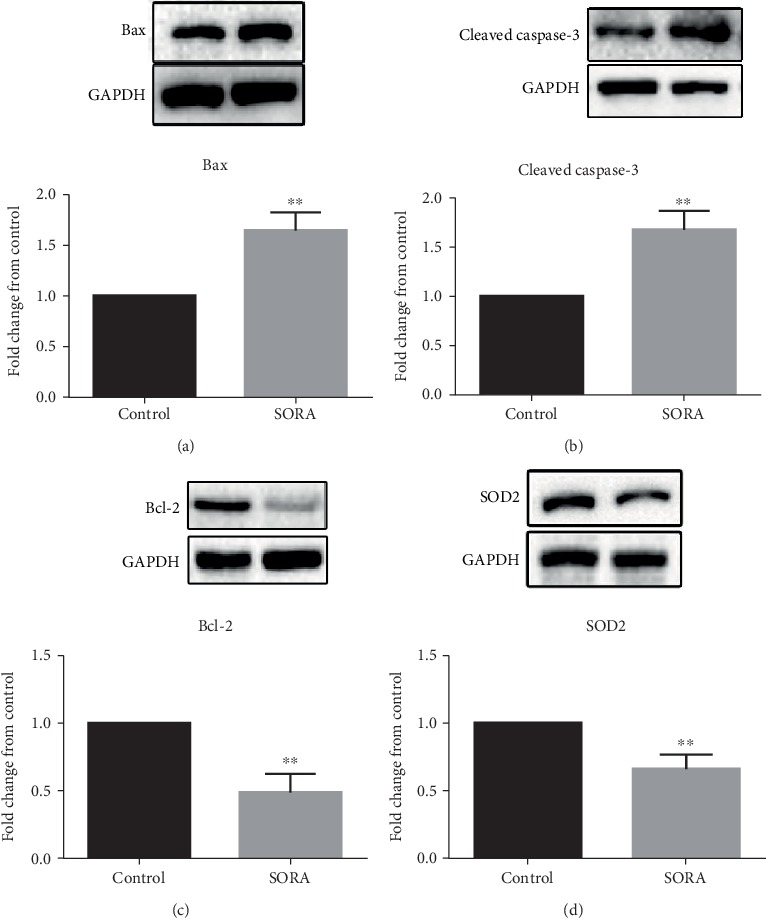
Protein expression analysis. Representative blots analysis of protein levels of (a) Bax, (b) cleaved Caspase-3, (c) Bcl-2, and (d) SOD2. Data are presented as mean ± SD. Where ^∗∗^*P* < 0.01. SORA: sorafenib; BAX: Bcl-2 Associated X; Bcl-2: B-cell lymphoma-2; SOD2: superoxide dismutase-2.

To further confirm our findings from gene expression studies, we used western blot analysis to determine levels of the proapoptotic proteins Bax and cleaved Caspase-3, the antiapoptotic protein Bcl-2, and the antioxidant protein SOD2. We found that SORA treatment remarkably induced Bax and cleaved Caspase-3 protein expression relative to the control group (1.6 folds and 1.7 folds, respectively) (Figures 3(a) and 3(b)). Furthermore, we observed considerable decreases in the expression of Bcl-2 and SOD2 proteins in SORA-treated rats (2 folds and 1.5 folds, respectively) (Figures 3(c) and 3(d)). These results further confirm that SORA treatment is associated with liver toxicity, as it activated oxidative stress and apoptotic pathways.

### 3.4. Sorafenib Attenuates the Activity of Antioxidant Enzymes

**Figure 4:**
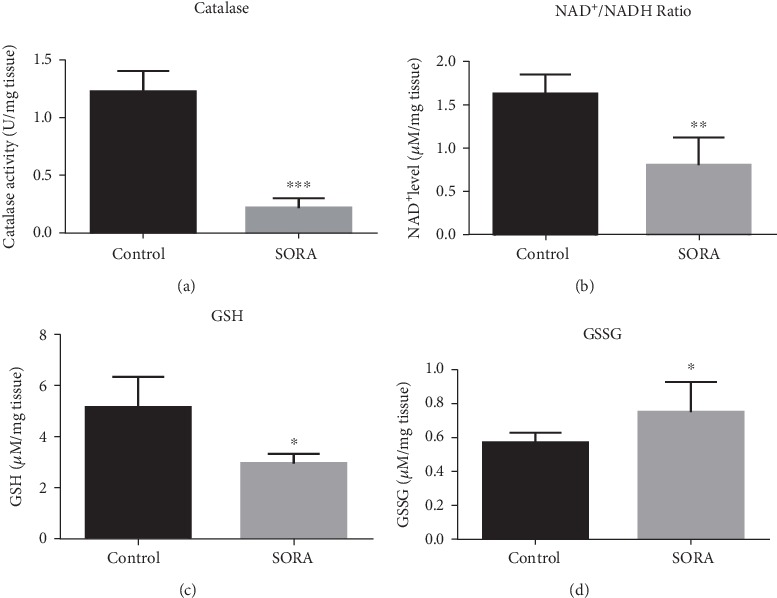
Biochemical assays. Liver tissues homogenates from control and SORA groups were obtained to analyze catalase activity (a), NAD^+^/NADH ratio (b), GSH (c), and GSSG (d). Data are presented as mean ± SD. Comparison between the control group and the sorafenib group is presented by ^∗^, where ^∗^*P* < 0.05, ^∗∗^*P* < 0.01, and ^∗∗∗^*P* < 0.001. SORA: sorafenib; NAD: Nicotinamide adenine dinucleotide; GSH: glutathione; GSSG: oxidized glutathione.

Several publications have reported that SORA treatment alters the activity of catalase, glutathione (GSH), oxidized glutathione (GSSG), and NAD^+^ [24–26]. Therefore, to further explore the toxic effects of SORA, we measured the enzyme activity of catalase and GSH along with the NAD^+^/NADH ratio. As shown in Figure 4, SORA administration significantly diminished the enzyme activity of catalase (5.5 folds) and the NAD^+^/NADH ratio (2 folds), as well as GSH (1.5 folds) (Figures 4(a)–4(c)). However, we demonstrated a marked increase in the levels of GSSG in SORA treated rats (1.3 folds) (Figure 4(d)). Together, these results support our previous findings that SORA treatment is associated with the induction of hepatotoxicity.

## 4. Discussion and Conclusion

In the current study, we studied the molecular mechanisms by which the TKI sorafenib (SORA) induces hepatotoxicity in rats. Our study mainly shows that at a dose of 30 mg/kg, SORA produces liver toxicity as evidenced by the reduction of antioxidant enzyme activities, elevation of hepatic enzymes, activation of the apoptotic pathway, and induction of oxidative stress markers. To the best of our knowledge, this is the first study to report the hepatotoxic effect of SORA using an *in vivo* model and to investigate the potential mechanisms underlying that toxicity. Future studies are needed to fully elucidate the molecular mechanisms underlying SORA-induced liver toxicity.

Drug-induced hepatotoxicity is a frequent and unpredictable potential risk during the treatment of numerous diseases, including gastrointestinal stromal tumor (GIST) [27, 28]. Sorafenib (SORA) is a well-known multikinase inhibitor that has the potential to inhibit cancer cell growth through its antiangiogenic effects and is used effectively in the treatment of advanced renal cell carcinoma and hepatocellular carcinoma. Several mechanisms by which SORA exerts antioncogenic effects have been reported, including the activation of JNK/p38-MAPK pathways and Bax translocation [13, 14, 16, 29]. Nonetheless, recent evidence has also demonstrated an association of SORA treatment with life-threatening hepatotoxicity in a 57-year-old male patient with GIST [28]. Furthermore, in a study that used rat liver mitochondria as a powerful *in vitro* model to test 31 approved kinase inhibitors, Zhang and his colleagues reported induction of hepatotoxicity by SORA treatment [15]. Of the 31 tested drugs, only sorafenib, pazopanib, and regorafenib showed mitochondrial toxicity when used at concentrations approximately near the maximum serum concentrations (Cmax). Their data collectively demonstrated three mitochondrial mechanisms that contributed to SORA-induced liver toxicity: mitochondrial swelling, decline in mitochondrial membrane potential, and inhibition of selective respiratory chain complexes. Although their study reported mitochondrial toxicity of SORA in an *in vitro* model, the exact mechanism through which SORA induces hepatotoxicity *in vivo* is yet to be considered.

Several reports have suggested alterations in liver enzymes such as ALP, AST, ALT, and urea and dysregulation of blood-associated proteins and lipids as reliable indicators of drug-mediated hepatotoxicity [30–34]. In the current study, SORA treatment significantly induced plasma levels of ALT and ALP, but not AST, while decreasing plasma levels of albumin, consistent with previously published reports [33, 35, 36]. Data from our current study demonstrated that SORA resulted in remarkable changes in cholesterol and LDL levels, and had no significant effects on triglyceride, HDL, or VLDL levels. The dramatic changes strongly indicate the ability of SORA to induce liver toxicity *in vivo*.

NF-*κ*B is a transcriptional factor whose inactive form is located in the cytoplasm and bound to the inhibitory subunit I-*κ*B. When a toxic agent is administered, reactive oxygen species (ROS) are produced, which further activates the NF-*κ*B pathway; this leads to the induction of apoptosis, which ultimately induces toxicity [37–39]. Although the mechanism of apoptosis involves several factors, it is believed that two protein families are primarily involved, the caspase family (especially caspase-3, 8, and 9) and the Bcl-2 family [39]. The caspase enzymes play essential roles in apoptosis, with caspase-3 being considered the most important as it contributes to several biochemical mechanisms that result in the cleavage of cytosolic and nuclear substances, condensation of chromatin, and DNA damage [40–44]. In order to understand the mechanism underlying SORA-induced hepatotoxicity, we analyzed different oxidative stress markers and the expression of antioxidant, proapoptotic, and antiapoptotic genes. Here, we report that SORA treatment significantly upregulated the expression of *NF-κB-p65* and the proapoptotic genes *Bax* and *cleaved Caspase-3*, whereas both gene and protein expressions of the antiapoptotic protein Bcl-2 and the antioxidant enzyme SOD2 were attenuated. Our results suggest that SORA induces liver toxicity through the activation of apoptotic pathways, which is in accordance with previously published papers [45–47].

An imbalance between antioxidants and the oxidative system of cells can result in a greater generation of oxidative free radicals, which can be removed from the biological system via enzymatic and nonenzymatic antioxidants [48–50]. Glutathione (GSH) plays a dual role; it can act as a nonenzymatic antioxidant that directly interacts with ROS through its thiol (–SH) group and as a cofactor during the enzymatic detoxification of ROS [51–53]. A reduced cytosolic NAD^+^/NADH ratio was reported in patients with fatty liver disease (NAFLD) and in animal models [54]. Any approach or treatment that increases cytosolic NAD^+^/NADH can help to improve NAFLD [54]. In the current study, we measured enzyme activities in order to further understand the mechanism of SORA toxicity. We demonstrated that antioxidant enzymes had diminished activities in SORA-treated animals compared to control animals, which further supports our findings that activation of oxidative stress and apoptotic pathways may contribute to the hepatotoxic effects of SORA. Furthermore, we found a significant decrease in the cytosolic NAD^+^/NADH ratio in liver homogenates of SORA-treated rats, which further suggests that SORA induces liver damage.

In conclusion, the current study is the first to demonstrate in an *in vivo* model both the potential hepatotoxic effect of SORA and the possible mechanism underlying this toxic effect. Further studies are required to fully understand the toxicity of SORA.

One of the limitations in the current study is that it does not measure the protein expression of NF-*κ*B-p65 to further validate the proposed mechanism. Furthermore, we acknowledge that measuring other direct oxidative stress markers and mitochondrial injury markers, such as H_2_O_2_, NADPH oxidase, and cytochrome c, would further support the proposed mechanism. However, a considerable part of our future study will focus on these issues to further confirm the hepatotoxic effect of SORA.

## Data Availability

The data used to support the findings of this study are included within the article.
